# Metal Ions Release from Welded Co—Cr Dental Alloys

**DOI:** 10.3390/ma16093398

**Published:** 2023-04-26

**Authors:** Andreja Carek, Ljerka Slokar Benić, Vatroslav Bubalo

**Affiliations:** 1 School of Dental Medicine, University of Zagreb, 10000 Zagreb, Croatia; acarek@sfzg.hr; 2 Faculty of Metallurgy, University of Zagreb, 44000 Sisak, Croatia; 3 Dubrava University Hospital, 10000 Zagreb, Croatia; vat.bub@inet.hr

**Keywords:** metal ion release, laser welding, tungsten inert gas welding, cobalt–chromium dental alloys, atomic absorption spectrometry, statistical analysis

## Abstract

Cobalt–chromium alloys (Co-Cr) are widely used in dentistry due to their excellent mechanical properties and corrosion resistance. Since prosthetic materials must be permanently stable in the oral cavity, it is very important to determine the release of ions from alloys in the oral cavity. In dentistry today, metals and alloys are mainly joined by laser and tungsten inert gas (TIG) welding. Therefore, in this work, the release of metal ions from six different Co-Cr alloys joined by these two welding methods was quantified to determine the effects of the welding method on an ion release. Static immersion tests, atomic absorption spectrometry and statistical analysis were performed for this purpose. The results showed that laser-welded alloys release a lower amount of metal ions compared to TIG-welded alloys.

## 1. Introduction

The common requirement for all prosthetic materials is that they must be permanently stable in the oral cavity, and therefore, it is very important to have quantified data on their release in the oral cavity. Considering that harmful processes take place in the oral cavity, such as dissolution and destruction of materials in saliva, wear and erosion by food, chewing and bacterial activity, it is important to evaluate the reactivity of materials in the oral cavity [1].

Due to their excellent properties, such as strength, stiffness, wear resistance, biological properties and corrosion resistance, Co-Cr alloys are widely used in dentistry for the manufacture of removable partial denture frameworks. Corrosion resistance results from Cr-based oxides formed on the surface, which contribute to biocompatibility over time [2,3,4]. The wide use of Co-Cr alloys in dentistry is due to good mechanical properties and economic reasons, and it is still not easy to find an effective alternative [5]. The wide range of different dental alloys on the market has led to uncontrolled and increased use of alloys in fixed prosthetics, which can lead to polymetallism in the mouth of prosthetic patients. Prosthetic works affect the tissues of the oral cavity in different ways. Because of their shape, they can promote plaque retention, make hygiene compliance impossible or press on the alveolar mucosa. They release ions, metal cations, which can cause low-voltage electrical currents with a burning sensation or metallic taste. More serious are allergic or cytotoxic effects locally or on distant organs. Prosthetic alloys increase the percentage of adhesion of oral bacteria due to their rough surface and surface tension in relation to the enamel. All this leads to changes in the electrical potential and surface energy. In addition, metals can have oligodynamic and even bacteriostatic effects on certain types of microorganisms [6]. Corrosion is the process of undesirable wear of restorative material under chemical and biological actions of the environment. Corrosion is always accompanied by a release of elements and a flow of electricity. Thanks to saliva as an electrolyte, electrochemical corrosion occurs in the moist environment of the oral cavity due to the different electropotential of the material. Saliva is a highly corrosive medium. The corrosiveness of saliva increases with decreasing saliva pH and increasing chloride concentration [1,7,8]. The corrosion of dental alloys in a biological medium is related to the biocompatibility of the alloy, its interaction with oral tissues and fluids. Despite the known composition, the release of the type and amount of ions from the alloys is unpredictable. Released metal ions and compounds resulting from corrosive changes may cause local irritation or systemic reactions, allergic sensitisation or toxic or carcinogenic effects [9,10,11].

In most dental alloy–electrolyte systems, corrosion at the surface itself is stopped by the formation of a surface oxide layer, which provides good protection against further corrosion, as is the case with cobalt–chromium alloys and titanium. By absorbing oxygen at the surface of the alloy, an oxide layer is formed that limits further dissolution of the other components of the alloy. Two different protective layers form in the oral cavity: oxide and sulphide. The sulphide layer is formed by a reaction between the alloy and sulphur from food and drink and causes dechlorination of the alloy and slows its further corrosive activity. Despite the formation of an oxide or sulphide layer, corrosion may continue in the mouth. Dental work that is subjected to strong masticatory forces during chewing will corrode more quickly than those where the frictional forces are not as pronounced. This phenomenon can be explained by the fact that the passivated oxide layer is removed from the surface of the alloy during chewing or even brushing. By removing a new layer, the surface of the workpiece wears more and more so that corrosion can reach the deeper layers of the alloy [12,13,14,15]. However, due to pitting, crevice corrosion and uniform corrosion, Co-Cr alloys release ions when implanted in the human body and become harmful to the patient [3,4]. Metal ions are released from dental alloys because they are exposed daily to pH changes in the mouth, mechanical stress and damage, temperature fluctuations, contact with food and drink and contact with microorganisms. The metal ions released by corrosion accumulate in the tissues of the oral cavity or in distant tissues and are excreted in the urine. Significantly higher amounts of cobalt and chromium were found in the saliva of patients with Co-Cr partial dentures when the dentures were in the mouth, with the amount of chromium released being almost twice that of cobalt. Older dentures released lower amounts of ions, suggesting that the surface of the alloy becomes passivated over time, reducing the degree of corrosion. Cr, Co, Ni, Fe and Zn ions were shown to be released from the Co-Cr alloy used to make the metal skeleton of partial dentures. Of these ions, Fe, Ni and Zn were not listed in the manufacturer’s declaration. The toxicity of Co-Cr alloys strongly depends on the degree of machinability of their surface: raw cast, untreated samples were found to be more toxic than sandblasted and polished samples. In addition, chromium and cobalt were found to elute more after brushing the prosthesis to maintain hygiene, suggesting that abrasion increases the corrosion of this relatively electrochemically passive alloy. A similar phenomenon has been observed with other dental alloys when brushed or mechanically abraded. It follows that the morphology of the surface of dental alloys, i.e., the type of casting, the degree of polishing and possible damage caused by brushing, abrasion or the chewing of hard foods are very important factors influencing the release of metal ions from the alloys [5,16,17,18].

In dentistry, joining is a prerequisite for joining metals and alloys. When it is necessary to repair damaged or broken metal structures of prosthetic works, one of the available techniques should be used. In the past, various techniques such as soldering, plasma or resistance welding were used. Today, laser welding with an Nd:YAG laser and TIG (tungsten-inert gas) welding are the most commonly used. In the TIG welding process, metal structures are joined by heating and melting by means of an arc formed between a nonconsumable electrode made of tungsten and the metal parts to be joined in a protective gas atmosphere. The shielding gas atmosphere is ensured by using argon or helium [19,20,21,22,23,24].

In the 1960s, Miaman introduced lasers into dentistry, and since then, their applications in dental practice have been continuously researched. Because pigmented tissue can absorb the laser’s wavelength very well, this type of laser is considered very effective for cutting and coagulating dental soft tissue. Pretreatments with the laser could also result in better shear adhesion of dental materials. Therefore, lasers are now an indispensable part of the dental industry [23,25,26,27].

In this work, six commercially available dental casting alloys were joined using Nd:YAG laser and TIG welding techniques. They were immersed in saliva before and after welding to characterise the effect of the joining process on corrosion stability by quantifying the cobalt, chromium and nickel ions released. The release of metal ions from dental alloys is very important as it plays an important role in the biocompatibility of alloys.

## 2. Materials and Methods

Six commercially available cobalt–chromium alloys with different chemical compositions and from different manufacturers were used in this study (Table 1). Three alloys—VI-COMP, (Austenal, Köln, Germany), Wirobond C (Bego, Bremen, Germany) and I- BOND NF (Interdent Celje, Slovenia)—are intended for the production of metal–ceramic works and two—Wisil M (Austenal, Köln, Germany) and I-MG (Interdent, Celje, Slovenia)—for denture frameworks, while one alloy, Brealloy F 400 (Bredent, Senden, Germany), is suitable for both purposes.

Castings of all alloys were produced by melting and casting in an induction furnace (Nautilus, Bego, Bremen, Germany). Nine specimens of each alloy were prepared for corrosion stability testing by the immersion test according to ISO 16744:2003 [28]. The specimens were divided into 3 groups as follows: in the first group, the specimens were joined using the Nd:YAG laser; in the second group, the specimens were joined using the TIG method; and the third group served as a control. The specimens of the first two groups were cut in the middle with a disc with intensive cooling and lubrication on an Accuto 2 cutter (Stuers, Rodovre, Denmark). The joining of specimens in the first group was realized using the Nd:YAG laser Hercules (Interdent, Celje, Slovenia) with a wavelength of 1064 nm and a beam diameter of 0.7 mm. The voltage used was 290 V, a pulse duration of 11 ms, a maximum pulse energy of 70 J and a spot weld overlap of 50–70%. Laser welding was carried out in an argon shielding gas environment (2–3 bar pressure). TIG welding was done using a Primotec Phaser Mx1 unit (Hafner, Pforzheim, Germany) according to the manufacturer’s instructions. The settings for TIG welding were as follows: welding current of 30 A, operating voltage of 20 V and pulse duration of 18 ms. The shielding gas environment for TIG welding was argon gas with a flow rate of 5 L/min and a pressure of 1 bar. The tungsten electrode was used at an angle of 45–60°, as consumption is minimised in this way. The third group consisted of unjoined specimens.

For the measurement of metal ion release, the specimens were cleaned in ethanol for 2 min in an ultrasonic bath, washed with distilled water and transferred to special test tubes. Then 1 mL of the immersion solution per cm^2^ surface area was poured into the test tubes so that the specimens were completely immersed in the solution. The immersion solution was prepared as follows: (10 ± 0.1) g of 90% lactic acid (90% C_3_H_6_O_3_, Ph. Eur., Fluka, Germany) and (5.85 ± 0.05) g of sodium chloride (NaCl_p.a._, Kemika, Zagreb, Croatia) were dissolved in 300 mL of ultrapure water (according to ISO 3696:1978) and diluted to (1000 ± 10) ml. The pH of the immersion solution was 2.3 ± 0.1. The test tubes were sealed to prevent evaporation of the solution and placed in a thermostat at 37 ± 1 °C for 7 days ± 1 h. Then, the mass concentrations of the released ions, i.e., the quantitative analysis of the release of Co, Cr and Ni ions from all specimens, were determined. PerkinElmer Aanalyst 800 atomic absorption spectrometer (AAS) with PerkinElmer “End Cup” graphite cuvettes was used for this. The parameters for carrying out the analysis AAS are listed in Table 2.

The temperature programme for carrying out the Cr, Co and Ni analysis is given in Table 3.

The mean and standard deviation are given to describe the elution of cobalt, chromium and nickel ions. Factorial MANOVA was used to test the influence of the alloy (A, B, C, D, E, F) and the group (welding type: laser, TIG and control group) on ion elution. Tukey’s test was used for posthoc multiple comparisons. The analysis was performed using the statistical package SAS on the Windows platform. All tests were performed with a significance level of α = 0.05.

In order to analyse the quality of the welds, the microstructure of the welded samples was examined with a light microscope (Olympus GX51 with CCD camera) and a scanning electron microscope (SEM Vega TS5136LS, Tescan, Brno, Czech Republic).

## 3. Results

**Figure 1:**
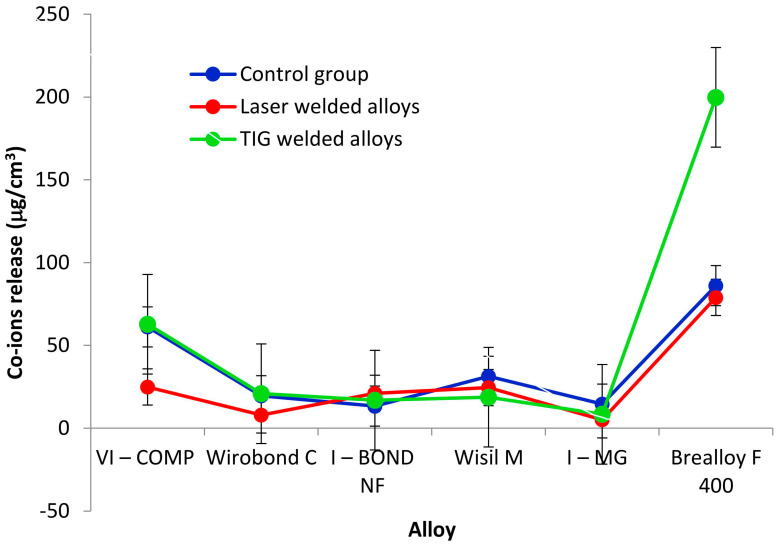
Cobalt ion release.

Ion release results are described by sample size, mean and standard deviation. The results of the release of cobalt ions are listed in Table 4 and shown in Figure 1.

**Figure 2:**
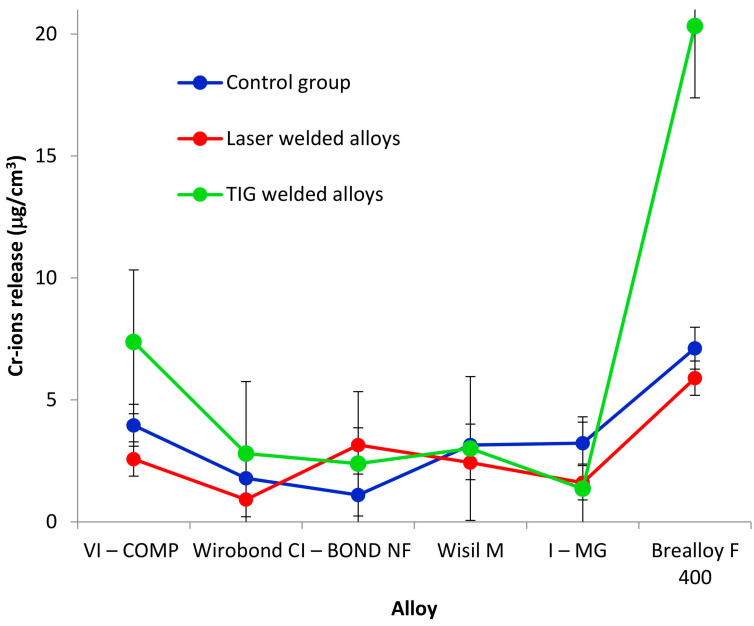
Chromium ion release.

The results of the release of chromium ions are listed in Table 5 and shown in Figure 2.

**Figure 3:**
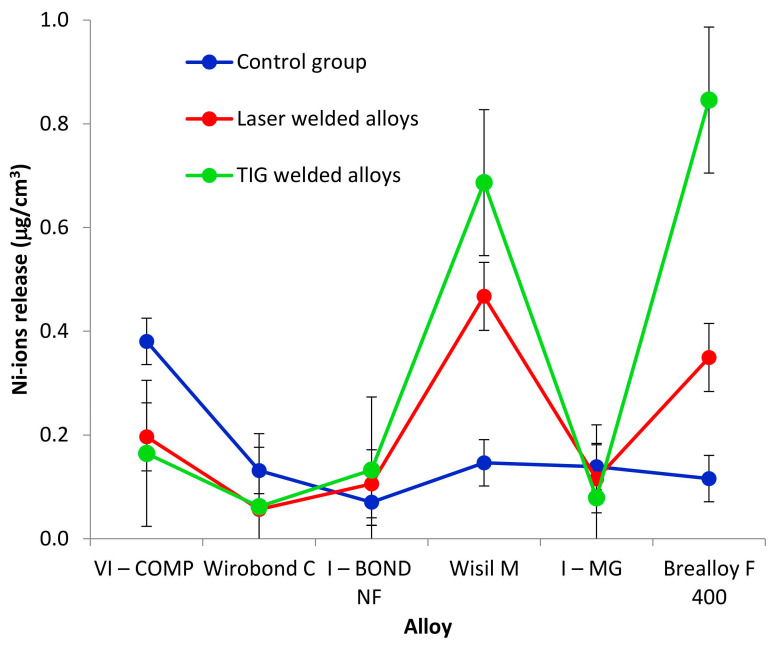
Nickel ion release.

The results of the release of nickel ions are listed in Table 6 and shown in Figure 3.

In the first step, the effects of the group and the alloy on the release of cobalt, chromium and nickel ions were investigated using the MANOVA test. It can be seen from Table 7 that the Wilks lambda is significant for both factors (group and alloy) and the interaction of group and alloy (*p* < 0.05 for Wilks’s lambda).

In the next step, the effects of the alloy and the group on the release of each metal ion were investigated using the ANOVA test. The results are shown in Table 8, Table 9 and Table 10.

From the results in Table 8, it can be seen that the group has no influence on the release of cobalt ions (*p* = 0.15; ANOVA test). The interaction of the group and the alloy is also not significant. Only the effect of the alloy is significant (*p* < 0.0001; ANOVA test).

The mean value of the released cobalt ions for all alloys is shown in Table 9. For the comparison of the cobalt ion release, the Tukey test for multiple comparisons was used. The release of cobalt ions is the highest for Brealloy F 400 (121.6 μg/cm^3^ on average) and is significantly different from the other five alloys. Among these five alloys, there is no statistically significant difference in the release of cobalt ions.

The analysis of the chromium ion release showed that the group and the alloy have an influence on the chromium ion release, while the interaction of the group and alloy is not significant (Table 10).

The influence of the group on the release of chromium ions is shown in Table 11. The release of chromium ions does not differ between the alloy welded with the laser and the control group (*p* > 0.05; Tukey test) or between the alloy welded with the TIG method and the control group (*p* > 0.05; Tukey test). A significant difference can only be seen between the release of chromium ions in the alloys welded with the laser (average 2.8 μg/cm^3^) and the alloys welded with the TIG method (average 6.2 μg/cm^3^).

The comparison of the alloys with regard to the release of chromium ions (Table 12) gave the same result as the comparison of the release of cobalt ions. The release of chromium ions is highest in Brealloy F 400 (average 11.1 μg/cm^3^) and is clearly different from the other five alloys. There is no statistically significant difference in the release of chromium ions between these five alloys.

The comparison of the release of nickel ions (Table 13) shows that, in addition to both factors, the interaction of the alloy and group is significant (*p* < 0.0001; ANOVA test).

Because of the significant interaction of alloys and groups, all 18 groups were compared using the Tukey test, comparing only the alloys for each group and the groups for each alloy separately. The result is shown in Table 14.

The release of nickel ions does not differ between the control group, the laser-welded alloys and the alloys welded with the TIG method: VI-COMP, Wirobond C, I-BOND NF and I-MG. For Wisil M, the release of nickel ions is significantly lower in the control group (average 0.15 μg/cm^3^), while there is no statistically significant difference in the release of nickel ions between the alloys welded with laser and alloys welded with TIG. For Brealloy F 400, the release of nickel ions is highest for the TIG-welded alloy (average 0.85 μg/cm^3^). Furthermore, for this alloy, the release of nickel ions does not differ between the control group and the laser-welded alloy.

In the control group, the release of nickel ions in VI-COMP is significantly higher than the release of nickel ions in I-BOND NF and Brealloy F 400, while the release of nickel ions for alloys VI-COMP, Wirobond C, Wisil M and I-MG do not differ. Furthermore, there is no statistically significant difference in the release of nickel ions for all alloys except for VI-COMP.

Among the alloys welded with the laser, the release of nickel ions in Wisil M is statistically significantly higher than the release of nickel ions in alloys VI-COMP, Wirobond C, I-BOND NF and I-MG, and the release of nickel ions in Brealloy F 400 is statistically significantly higher than the release of nickel ions in Wirobond C. There is no statistically significant difference between the alloys VI-COMP, Wirobond C, I-BOND NF and I-MG as in the alloys VI-COMP, I-BOND NF, I-MG and Brealloy F 400. There is no statistically significant difference between Wisil M and Brealloy F 400.

In TIG-welded alloys, there is no difference between Wisil M and Brealloy F 400 or between VI-COMP, Wirobond C, I-BOND NF and I-MG. The release of nickel ions in Wisil M and Brealloy F 400 is significantly higher than the release of nickel ions in VI-COMP, Wirobond C, I-BOND NF and I-MG.

**Figure 4:**
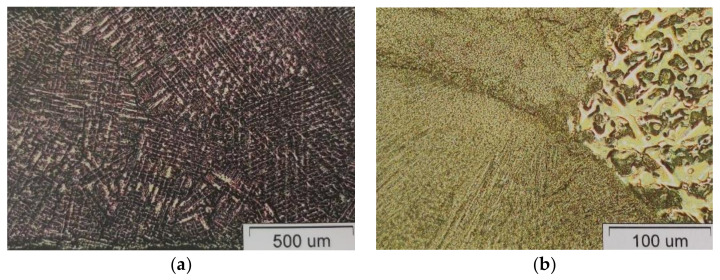
Light micrographs of Wisil M. (**a**) Casting sample; (**b**) TIG weld.

To gain better insight into joint quality and the effects of the joint method on ion release, a microstructure analysis was carried out. Figure 4 shows light micrographs of the alloy for dental frameworks, I-MG, which released the least amount of metal ions. These micrographs show the dendritic microstructure of the castings. In the microstructure of the welded sample, coarse dendritic grains, a fine-grained weld zone and a narrow heat-affected zone can be seen. No defects, such as porosity or cracks, are visible.

**Figure 5:**
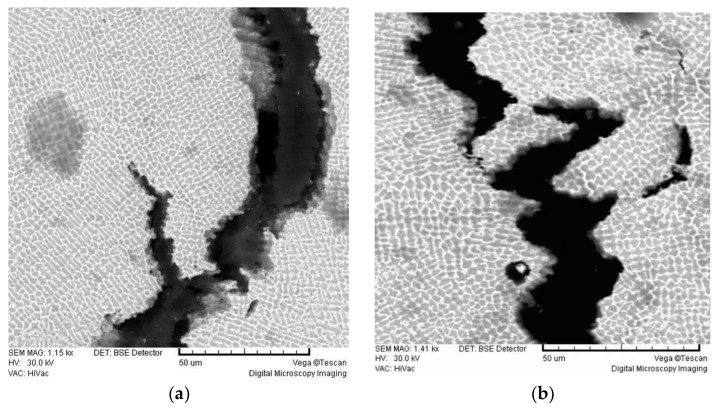
SEM micrographs of Brealloy F 400. (**a**) Laser weld; (**b**) TIG weld.

Figure 5 shows SEM micrographs of the alloy Brealloy F 400, which is intended for both metal–ceramic and dental frameworks and releases the largest amount of metal ions in both processes, both in the cast state and after welding. A crack can be seen extending longitudinally along the specimen through the centre of the weld material. It is obvious that the crack has an intergranular character, i.e., it spreads along the grain boundaries. This indicates that it is caused by stresses in the material, i.e., internal stresses most likely caused by large temperature differences during the welding process.

**Figure 6:**
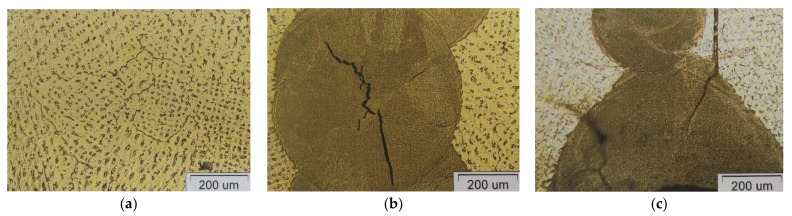
Light micrograph of VI-COMP. (**a**) Casting; (**b**) Laser weld; (**c**) TIG weld.

Figure 6 shows light micrographs of the VI-COMP alloy, which, of the three alloys for metal–ceramic works, released the highest amount of metal ions.

The base material is relatively coarse-grained. Defects such as cracks were found in the welds. In the laser-welded sample, an irregular large crack through the weld and several small cracks within the weld are visible. The irregularity or branching of the crack indicates that it is a stress crack. In the TIG-welded sample, the extremely fine-grained structure of the weld material in relation to the base material can be seen. In addition, a very narrow heat-affected zone is visible. In addition, two cracks are visible at the root of the weld, spreading from the centre of the weld and penetrating into the base material.

**Figure 7:**
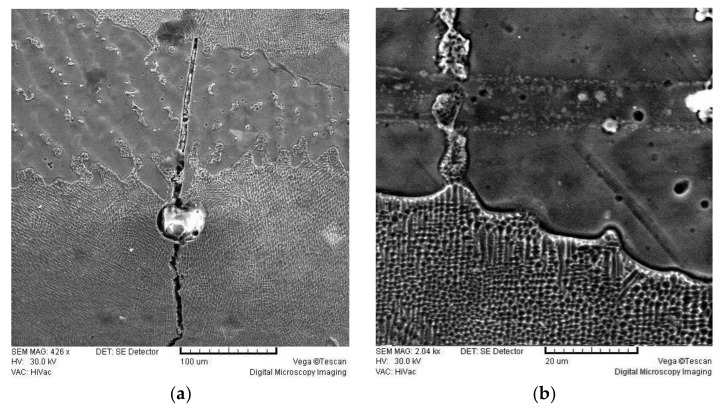
SEM micrographs of I-BOND NF. (**a**) Laser weld; (**b**) TIG weld.

Figure 7 shows the SEM micrographs of the alloy I-BOND NF.

The SEM micrographs of Brealloy F 400, which were intended for both purposes and showed the highest release of metal ions, show many defects, such as cracks and pores. In the laser-welded sample, a large crack of varying width, depth and extent can be seen along with some inclusions. In the TIG-welded samples, a dendritic structure and a narrow heat-affected zone can be seen.

## 4. Discussion

The corrosion of dental alloys and the release of metal ions that can affect health occur due to the complex oral environment [29]. The release of ions from metals in the presence of water, aqueous salt solutions and acids results in electrochemical corrosion, which has greater clinical significance as it occurs in the moist environment of the oral cavity under salivary electrolytes containing dissolved anions and cations of dissociated compounds.

In this work, samples of castings made of six different cobalt–chromium alloys were divided into three groups. The first group served as control and contained samples that were not welded. In the second group were samples that were welded by laser, while in the third group were samples welded by TIG procedure.

In clinical practice, alloy castings often need to be joined, either for expedient connections or to repair fractures. When comparing soldering, as the oldest and longest-used joining method in dental prosthetics, with welding, welding is increasingly preferred. The reason for this is that in welding, the heat input is limited to the welding point, which allows working in close proximity to the ceramic or acrylic part of the structure, even in the patient’s mouth.

Properly welded joints contribute to the passivation of the alloy, thereby increasing the alloy’s resistance to corrosion compared to soldering, where the solder joint is the most unstable point of corrosion. Different types of alloys, such as high-quality gold alloys and Co-Cr alloys, can be welded together but not soldered. Welding processes are less time-consuming compared to soldering [30]. Dielert and Kassenbacher compared the quality of joints made by soldering, laser and plasma welding. Based on their results, they conclude that welding is superior to soldering [31].

Here, all samples were tested for the release of cobalt, chromium and nickel ions after immersion in the lactic acid–sodium chloride solution (pH = 2.3). Although the manufacturers emphasise the absence of nickel in their alloys in the declaration, the tests in this work detected the presence of nickel, albeit in a low proportion in all alloys. A higher release was detected in the Wisil M and Brealloy F 400 alloys. When comparing the welding methods, the release of nickel ions is higher for arc-welded samples (TIG). The rule is that the manufacturer does not have to declare the content of elements in an amount of less than 2 wt.%. However, if an element is suspect in any way, such as nickel for allergies, its proportion must be ticked. The claim that cobalt–chromium alloys are completely nickel-free would be misleading and can be understood from the market side. In prosthetic work made of Co-Cr alloys weighing 10 grammes (for partial dentures), the entire structure contains a maximum of 0.07 g of nickel. The nickel release is about 0.00003 mg/cm^2^ in the first week and then decreases steadily. If this is compared with the daily dietary intake of 0.19–0.90 mg of nickel in the body, the aforementioned amount of nickel in alloys is negligible. The release of ions from dental alloys is inevitable, as it is difficult to find an alloy that is completely stable in the body and shows no signs of biodegradation. However, nickel from dental alloys has been shown to accumulate in cells over a long period of time and can have a harmful effect on living cells at higher concentrations [32,33]. The higher release of cobalt ions was found in TIG-welded Brealloy F 400, which has the highest cobalt content in its composition. However, according to Wataha [10], the alloy does not necessarily release elements in proportion to its chemical composition.

In most cases, the results presented here showed that the lowest release of cobalt, chromium and nickel ions was found in laser-welded samples, even lower than in unwelded samples. This indicates a favourable effect of laser welding on the samples. Dobberstein [34] indeed mentions good mechanical properties and increased corrosion resistance as the main advantages of laser welding of Co-Cr alloys. The same opinion is held by Zupančić [35], who demonstrated in his work that laser welding of Co-Cr alloys is corrosion resistant, but its strength is limited due to the low penetration depth of the weld.

When looking at the results obtained, different stabilities of the released elements of interest can be seen. For example, the amount of cobalt ions released was 10 times greater than that of chromium. Furthermore, Wataha and Lockwood [13] studied the release of ions from eight different dental alloys (precious and base alloys) over a period of 10 months and concluded that the different elements in the alloy have different stability.

Finally, comparing the welding methods used in this study, the release of cobalt and chromium ions is found to be higher in the arc-joined samples, and as far as the type of alloy is concerned, the release is highest in the cast Brealloy F 400 samples.

The therapeutic effect of all castings depends on the appearance of the microstructure, which is determined by a number of laboratory procedures, the quality of the alloy before casting, the investment material, the melting temperature, the cooling rate and the surface treatment. Castings of base alloys usually crystallise dendritically, which is a consequence of the cooling rate and the proportion of several components with different melting points. Compared to the base material, the weld material has a much finer-grained dendritic structure. Investigations of the microstructure of the tested samples showed that the Co-Cr alloy castings crystallise dendritically and coarsely. In contrast to the base material, the weld material exhibits an extremely fine-grained microstructure regardless of the joining process. Stress cracks of different shapes and propagation directions can be seen at the resulting joints. The requirement that 80% of the previous weld should be covered by laser joining and at least 50% by TIG is not fulfilled in all specimens, which could be one of the reasons for the appearance of cracks. Their appearance, location and distribution indicate that they are cold cracks caused by internal stresses during the cooling of the material.

## 5. Conclusions

The investigation of the release of cobalt, chromium and nickel ions from six cobalt–chromium alloys of different chemical compositions, which were welded with the laser and the TIG methods, proves the connection between the joining process and corrosion resistance. Immersion tests proved the release of cobalt, chromium and nickel ions, although nickel was not declared in any of the alloys tested. Compared to arc welds, laser welds are more corrosion resistant. Among the alloys for metal–ceramic works, Wirobond C is the most stable, while among the alloys for denture frameworks, alloy I-MG proved to be the most stable. From the comparison between the quality of the joint and the amount of metal ions released, it appears that the release is lower in samples with a better quality of joint.

## Figures and Tables

**Table 1:** Chemical composition of the alloys.

Tradename	Composition (wt. %)											
	Co	Cr	Mo	Mn	Si	N	C	Nb	W	Fe	Ni	Ce
VI-COMP	61.0	32.0	5.5	0.7	0.7	/	/	/	/	/	/	/
Wirobond C	61.0	26.0	6.0	/	1.0	/	0.02	/	5.0	0.5	/	0.5
I-BOND NF	63.0	24.0	3.0	/	1.0	/	/	1	8.0	/	/	/
Wisil M	63.1	28.0	6.0	1.0	0.8	/	0.50	/	0.6	/	/	/
I-MG	62.5	29.5	5.5	0.6	1.4	0.2	0.30	/	/	/	/	/
Brealloy F 400	64.7	29.0	5.0	0.4	0.5	/	0.40	/	/	/	/	/

**Table 2:** General parameters for measurements with the atomic absorption spectrometer.

Parameter/Element	Co	Cr	Ni	Co
Wavelength, nm	242.5	357.9	232.0	242.5
Spectral bandwidth, nm	0.2	0.7	0.2	0.2
Matrix modifier	Mg (NO_3_)_2_	Mg (NO_3_)_2_	Mg (NO_3_)_2_	Mg (NO_3_)_2_
Acidified or diluted	0.2% HNO_3_	0.2% HNO_3_	0.2% HNO_3_	0.2% HNO_3_
Characteristic mass, pg	17.0	7.0 pg	17.0 pg	17.0

**Table 3:** Temperature programme for the elemental analysis by AAS.

Stage	Temperature, °C			Time to Reach the Set Temperature, s			Holding Time at the Set Temperature, s			Argon Flow through the Cuvette, mL/min		
	Co	Cr	Ni	Co	Cr	Ni	Co	Cr	Ni	Co	Cr	Ni
Drying 1	110	110	110	1	1	1	20	20	30	250	250	250
Drying 2	130	130	130	5	5	15	30	30	30	250	250	250
Pyrolysis	1400	1300	1100	10	10	10	20	20	20	250	250	250
Atomization	2400	2300	2400	0	0	0	5	5	5	0	0	0
Cleaning	2450	2400	2500	1	1	1	2	2	5	250	250	250

**Table 4:** Descriptive statistics for the release of cobalt ions.

Group	Alloy	N	Mean	SD
	VI-COMP	3	61.1	18.3
	Wirobond C	3	19.6	16.8
Control	I-BOND NF	3	13.3	11.7
	Wisil M	3	31.3	21.1
	I-MG	3	14.5	1.8
	Brealloy F 400	3	86.1	73.3
	VI-COMP	3	24.9	5.5
	Wirobond C	3	8.0	0.8
Laser-welded	I-BOND NF	3	21.0	18.2
	Wisil M	3	24.5	17.4
	I-MG	3	5.0	0.4
	Brealloy F 400	3	79.0	21.9
	VI-COMP	3	62.7	6.8
	Wirobond C	3	20.8	10.9
TIG-welded	I-BOND NF	3	16.9	9.9
	Wisil M	3	18.7	5.2
	I-MG	3	8.3	1.4
	Brealloy F 400	3	199.8	150.7

**Table 5:** Descriptive statistics for the release of chromium ions.

Group	Alloy	N	Mean	SD
	VI-COMP	3	4.0	1.4
	Wirobond C	3	1.8	1.5
Control	I-BOND NF	3	1.1	0.8
	Wisil M	3	3.2	2.0
	I-MG	3	3.2	3.6
	Brealloy F 400	3	7.1	3.9
	VI-COMP	3	2.6	0.3
	Wirobond C	3	0.9	0.3
Laser-welded	I-BOND NF	3	3.2	2.8
	Wisil M	3	2.4	1.3
	I-MG	3	1.6	0.9
	Brealloy F 400	3	5.9	2.2
	VI-COMP	3	7.4	0.7
	Wirobond C	3	2.8	1.4
TIG-welded	I-BOND NF	3	2.4	1.3
	Wisil M	3	3.0	0.8
	I-MG	3	1.4	0.4
	Brealloy F 400	3	20.3	15.5

**Table 6:** Descriptive statistics for the release of nickel ions.

Group	Alloy	N	Mean	SD
	VI-COMP	3	0.38	0.02
	Wirobond C	3	0.13	0.10
Control	I-BOND NF	3	0.07	0.00
	Wisil M	3	0.15	0.03
	I-MG	3	0.14	0.05
	Brealloy F 400	3	0.12	0.04
	VI-COMP	3	0.20	0.08
	Wirobond C	3	0.06	0.01
Laser-welded	I-BOND NF	3	0.11	0.03
	Wisil M	3	0.47	0.14
	I-MG	3	0.12	0.03
	Brealloy F 400	3	0.35	0.07
	VI-COMP	3	0.16	0.04
	Wirobond C	3	0.06	0.01
TIG-welded	I-BOND NF	3	0.13	0.01
	Wisil M	3	0.69	0.30
	I-MG	3	0.08	0.03
	Brealloy F 400	3	0.85	0.15

**Table 7:** MANOVA test.

Factor	Wilks’ Lambda	*p*
Group	0.46	0.0001
Alloy	0.11	<0.0001
Interaction	0.13	<0.0001

**Table 8:** ANOVA test for cobalt ions release.

Source of Variation	DF	SS	MSS	F	*p*
Group	2	6915.3	3457.7	2.03	0.15
Alloy	5	81183.8	16236.8	9.51	<0.0001
Interaction	10	24183.5	2418.4	1.42	0.21
Error	36	61445.2	1706.8		
Total	53	173727.8			

**Table 9:** ‘Posthoc’ test for cobalt ions.

Alloy	Mean
VI-COMP	49.6 ^a^
Wirobond C	16.1 ^a^
I-BOND NF	17.1 ^a^
Wisil M	24.8 ^a^
I-MG	9.3 ^a^
Brealloy F 400	121.6

^a^—there is no significant difference between alloys with the same letter.

**Table 10:** ANOVA test for chromium ion release.

Source of Variation	DF	SS	MSS	F	*p*
Group	2	121.5	60.8	3.67	0.035
Alloy	5	574.7	114.9	6.94	0.0001
Interaction	10	318.6	31.9	1.92	0.07
Error	36	595.8	16.6		
Total	53	1610.7			

**Table 11:** ‘Posthoc’ test for chromium ions.

Group	Mean
Control	3.4 ^ab^
Laser-welded alloys	2.8 ^a^
TIG-welded alloys	6.2 ^b^

^a,b^—there is no significant difference between groups with the same letter.

**Table 12:** Posthoc’ test for chromium ions.

Alloy	Mean
VI-COMP	4.6 ^a^
Wirobond C	1.8 ^a^
I-BOND NF	2.2 ^a^
Wisil M	2.9 ^a^
I-MG	2.1 ^a^
Brealloy F 400	11.1

^a^—there is no significant difference between groups with the same letter.

**Table 13:** Result of the ANOVA test for nickel ions release.

Source of Variation	DF	SS	MSS	F	*p*
Group	2	0.25	0.13	14.04	<0.0001
Alloy	5	1.23	0.25	27.02	<0.0001
Interaction	10	1.13	0.11	12.40	<0.0001
Error	36	0.33	0.01		
Total	53	2.93			

**Table 14:** ‘Posthoc’ test for nickel ions.

	Control Group	Laser-Welded Alloys	TIG-Welded Alloys
Alloy	Mean	Mean	Mean
VI-COMP	0.38 ^aA^	0.20 ^cdA^	0.16 ^gA^
Wirobond C	0.13 ^abB^	0.06 ^cB^	0.06 ^gB^
I-BOND NF	0.07 ^bC^	0.11 ^cdC^	0.13 ^gC^
Wisil M	0.15 ^ab^	0.47 ^eD^	0.69 ^fD^
I-MG	0.14 ^abE^	0.12 ^cdE^	0.08 ^gE^
Brealloy F 400	0.12 ^bF^	0.35 ^deF^	0.85 ^f^

^a–g^—there is no significant difference between groups with the same letter. ^A–F^—there is no significant difference between groups with the same letter.

## Data Availability

Not applicable.
